# Maintenance of mitochondrial DNA copy number is essential for osteoclast survival

**DOI:** 10.1186/ar3650

**Published:** 2012-02-09

**Authors:** Tsuyoshi Miyazaki, Shuuichi Mori, Kazuhiro Shigemoto, Nils-Goran Larsson, Takeshi Nakamura, Shigekaki Kato, Tomoki Nakashima, Hiroshi Takayanagi, Sakae Tanaka

**Affiliations:** 1 Department of Geriatric Medicine, Tokyo Metropolitan Geriatric Hospital and Institute of Gerontology, Tokyo 173-0015, Japan; 2 Division of Metabolic Diseases, Karolinska Institute, Stockholm, Sweden; 3 Institute of Molecular and Cellular Biosciences, The University of Tokyo, Tokyo 113-0032, Japan; 4 Department of Cell Signaling, Graduate School of Medical and Dental Sciences, Tokyo Medical and Dental University, Tokyo 113-8549, Japan; 5 Department of Orthopaedic Surgery, Faculty of Medicine, The University of Tokyo, Tokyo 113-0033, Japan

## Background

There is accumulating evidence that osteoclasts, the primary cells responsible for bone resorption, are involved in bone and joint destruction in rheumatoid arthritis. Bone resorption is highly regulated by mature osteoclast function as well as osteoclastogenesis. The life span of mature osteoclasts is relatively short both *in vitro *and *in vivo*, and once differentiated, they rapidly die in the absence of supporting cell or growth factors. Mitochondria is known as powerhouse of cell because they generate most of the cell's supply of adenosine triphosphate (ATP), used as a source of chemical energy. In addition to supplying cellular energy, mitochondria are involved in a range of other processes, such as signaling, cellular differentiation, cell growth, and cell death. Transcription and replication of mitochondrial DNA (mtDNA) are important steps in mitochondrial biogenesis and mitochondrial transcription factor A (Tfam) is essential for mtDNA transcription and replication. However, the functional significance of mitochondria has not been established in osteoclastic bone resorption.

## Materials and methods

To address this question, we generated osteoclast-specific Tfam conditional knock-out (cKO) mice by mating Tfam^fl/fl ^mice with cathepsin K-Cre transgenic mice, in which the Cre recombinase gene is knocked into the cathepsin K locus and specifically expressed in mature osteoclasts. The in vivo effects of Tfam deficiency on bone metabolism were examined by histological and histomorphometric analysis. The survival and bone-resorbing activity of Tfam cKO osteoclasts were determined by in vitro survival assay and pit formation assay, respectively.

## Results

The expression level of Tfam, mtDNA copy number, and cellular ATP level were markedly reduced in osteoclasts derived from Tfam cKO mice. The body size of Tfam cKO mice was smaller than that of the control mice, although trabecular bone volume remained unchanged by Tfam deficiency. However, histological sections of proximal tibia and lumbar spine of Tfam cKO mice showed significantly decreased osteoclast number. Interestingly, Tfam cKO osteoclasts exhibited increased bone-resorbing activity in spite of their pro-apoptotic tendency.

## Conclusions

This study demonstrates that Tfam cKO osteoclasts exhibited increased bone resorption with accelerated apoptosis, indicating that there may be an inverse correlation between osteoclast survival vs bone resorption. Further investigation of mitochondria in bone-resorbing osteoclasts will give us new insights into the molecular mechanism regulating bone homeostasis.

